# Reconfigurable high-order mode pass filter for mode-division multiplexing

**DOI:** 10.1016/j.heliyon.2022.e11706

**Published:** 2022-11-18

**Authors:** Prapty Saha, Oruni Aminul, Md. Atiqur Rahman, Md. Shah Alam, Kazi Tanvir Ahmmed

**Affiliations:** Department of Electrical and Electronic Engineering, University of Chittagong, Chittagong, 4331, Bangladesh

**Keywords:** Mode filter, Mode converter, Planar waveguide, MZI device

## Abstract

In mode division multiplexing (MDM) optical system, the mode filter has become an inseparable part to reduce modal crosstalk and transmit the desired modes unabatedly. As filtering out lower-order mode is difficult, here we propose a reconfigurable structure of a higher-order mode pass filter consisting of two tunable mode converters and a directional coupler (DC) in a three-mode planar waveguide platform. By switching the working states of the mode converters, the structure can also be used as a fundamental mode (TE_0_) pass filter and hence dynamic output signals can be achieved. For the second-order mode (TE_2_) transmission, the simulated excess loss is ∼0.61 dB at 1.550 μm and the extinction ratio remains ≥24 dB (power ratio of TE_0_ & TE_2_ Launch) and ≥25 dB (power ratio of TE_1_ & TE_2_ Launch) at the entire C-band (1.530–1.565 μm). The device has negligible polarization dependence and hence the TM polarization exhibits similar results.

## 1 Introduction

Optical interconnects have been proved successful to cope with the increasing demand of network traffic concerning the copper cables and metallic waveguides because of their low latency, low power consumption, and broadband nature [1]. However, the transmission capacity of an optical fiber is not limitless thence various innovative technologies have been introduced to stretch the capacity limit of a fiber. Among all these technologies, the mode-division multiplexing (MDM) on the silicon-on-insulator (SOI) platform is one of the most promising alternatives due to its compact size, high performance, and compatibility with the complementary-metal-oxide semiconductor (CMOS) fabrication process [2]. In the MDM technique, the information capacity is increased seamlessly by shifting from single-mode to multi-mode where each mode transmits data as an independent channel [3, 4, 5]. Based on the SOI platform various types of multi-mode passive devices like mode multiplexer (MUX)/de-multiplexer (De-MUX) [6, 7], mode converters [8, 9], mode selective switches [10, 11, 12], mode splitter [13] have been reported to develop the on-chip MDM technique. At the same time, a planar lightwave circuit (PLC)-based platform is being used commonly because PLC devices have high integration capability, and more robustness due to their geometry, high compatibility, and fabrication flexibility.

MDM technique can be made more feasible by filtering out the undesired modes using a mode filter in a similar way the wavelength filter is used in wavelength-division multiplexing systems (WDM) [14]. As the lower order modes are difficult to filter out from the fiber/waveguide for their good confinement capability, the mode filter has become an essential device in the MDM system. However, there have been several numbers of researches reported on higher-order mode (HOM) pass filters in embedded planar waveguide platforms. Some of the designs are demonstrated based on optically resonant devices such as 1D photonic crystals [15] and sub-wavelength gratings (SWG) [16, 17, 18], some are based on materials with special optical properties [19, 20, 21, 22, 23], inverse design structure [24], multimode interference [25] and mode converters incorporated to Mach-Zehnder interferometer (MZI) [26, 27].

Guan et al. [15] designed the waveguide as a Bragg grating and SWG for the TE_0_ and TE_1_ modes respectively where the TE_1_ mode can pass through the SWG by acting as a Bloch mode and the TE_0_ mode is blocked. This device has an insertion loss of 1.3 dB and operates in a limited bandwidth for the Bragg condition. In another approach, a two-mode blocking filter was demonstrated by Yu He et al. [16] using SWG based contra-directional coupler. The insertion loss of this device is ≤2.30 dB which can be reduced by increasing the distance between the two waveguides but this limits the operational bandwidth. Phase-shifted long-period grating was used in a three-mode waveguide by Huang et al. [17] to reject the fundamental mode. Though this device has a broad operational bandwidth of >140 nm, this device is limited to the insertion loss of >1.50 dB. Recently, Jiang et al. [18] proposed a HOM pass filter using cascaded plasmonic bridged SWGs where TM_0_ mode is absorbed and radiated out while TM_1_ can pass through the device. Compared to other SWG-based approaches this design structure has a lower insertion loss of 0.63 dB but this device was designed only for the TM polarization. In 2016, a HOM pass filter design was proposed based on Hyperbolic Meta-Materials (HMM) [19] and VO_2_ [20] with special optical properties. Though the HMM-based designed structure [19] can work as a mode selective HOM pass filter yet there is no report on the existence of hyperbolic material in nature for 1550 nm wavelength. Some more approaches to the HOM pass filter were demonstrated using graphene on a polymer waveguide platform [21, 22, 23]. These structures can be operated in a wide bandwidth but they suffer from high optical losses. G. You et al. [24] proposed an inverse design structure-based ultra-compact and broadband HOM-pass filter and achieve a compact footprint, a low insertion loss<0.26 dB though it is nontrivial to be fabricated. The most prominent HOM pass filter designs were proposed and demonstrated using a mode converter incorporated with MZI for both passive and tunable configurations in [26] and [27]. The basic idea was to convert the fundamental mode into first-order mode and filtered it out using an adiabatic taper. Although this approach is efficient for achieving a low insertion loss of ≤0.52 dB and a high extinction ratio of 37 dB [26], the structure was designed for only two modes. The operation of some of these devices is confined to either transverse magnetic (TM) or transverse electric (TE) modes. Moreover, some structures are focused on blocking only one specific high-order mode, and multiple-mode filtering by these structures requires the cascading of various mode blocking filters.

In this paper, we propose a low loss and integrate broadband higher-order mode pass filter, capable of handling three modes, using a mode converter incorporated with MZI in the PLC platform. The structure consists of two switchable mode converters and two-directional couplers. The directional couplers are designed to couple only the fundamental mode while the other modes will be radiated out. The switching ability of the mode converters has added diversity to this configuration by increasing the flexibility of the mode conversion process. The same structure can be used as both higher-order-mode pass filter and fundamental-mode pass filter separately by tuning the “ON-OFF” states of the mode converters. Moreover, by tuning the second mode converter the mode of the output signal can be exchanged between fundamental and second-order mode optionally.

## 2 Device structure and operation principle

**Figure 1:**
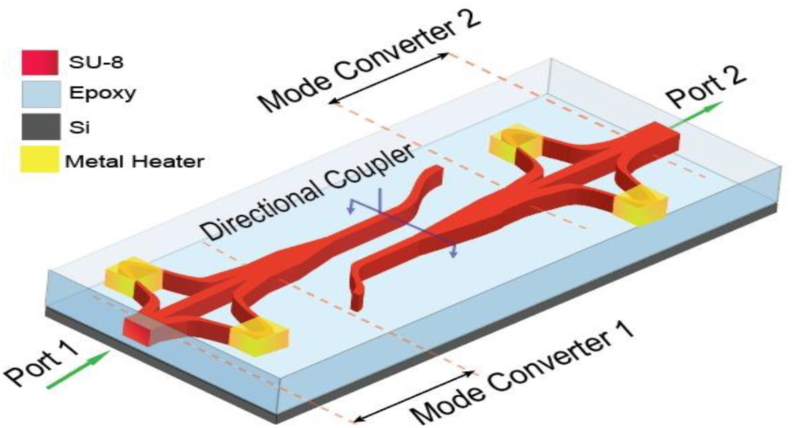
Schematic configuration of reconfigurable HOM pass filter.

The schematic configuration of the proposed three-mode, higher-order-mode pass filter is illustrated in Figure 1. The structure consists of two-mode converter devices to convert the fundamental mode to the second-order mode and vice versa while the first-order mode remains unchanged and a directional coupler (DC). Two individual tapers have connected the two ports of the directional coupler to the mode converters. The directional coupler rejects the higher-order modes and only couples the fundamental mode. The first mode converter converts the launched second-order mode (TE_2_) at Port 1 to the fundamental mode (TE_0_) and then the converted TE_0_ mode appears at the primary waveguide of the DC through the first tapper. As the DC is designed to couple the TE_0_ mode so the TE_0_ mode is easily coupled at the secondary waveguide of the DC and through another taper appears at mode converter 2 and is finally converted back to TE_2_ mode. Again, when the TE_0_ mode or the TE_1_ mode is launched at Port 1, no light appears at Port 2. These two modes are radiated out to the cladding. The launched TE_0_ mode is converted to TE_2_ mode and the launched TE_1_ mode remains unchanged and when they appear at the primary waveguide of the DC, they are radiated out as the DC only couples the TE_0_ mode. If all the three modes (TE_0_, TE_1_ & TE_2_) are launched at the same time, only the TE_2_ mode will be passed through the filter and the rest of the modes will be blocked. Thus, the structure works as a higher-order mode pass filter.

**Figure 2:**
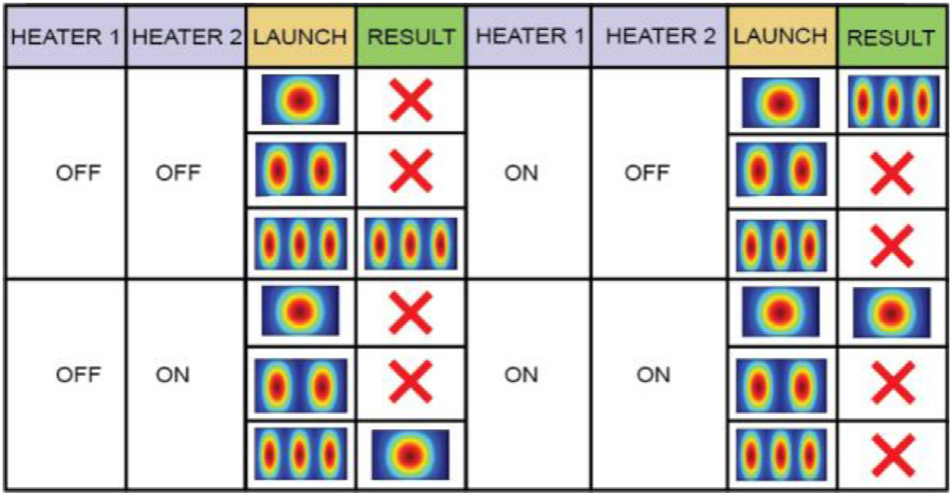
Switching Matrix of the proposed high order mode pass filter.

The structure can also be used as a fundamental mode pass filter. For this operation, we have added a phase-shifter forming heater on two unbalanced arms of the mode-dependent MZI structure. By driving the phase shifter, we can tune the “ON-OFF” states of the mode converters. When both mode converters are in the “OFF” state, the whole structure works as a HOM-pass filter, and when in the “ON” state, it works as a fundamental-mode-pass filter. This filtering principle is also applicable for TM polarization. We can assume the heaters of the first mode converter as “Heater 1”, the heaters of the second mode converter as “Heater 2”. Here, “0” represents the “OFF” state of the heaters and “1” represents the “ON” state of the heaters. For different switching states of the heaters, the resulted outputs are shown in Figure 2.

The structure of the mode converter is designed using an unbalanced MZI and two Y junctions where the stem of the Y junction supports three modes: the fundamental mode (TE_0_), the first-order mode (TE_1_) and the second-order mode (TE_2_). But the arms of the Y junction and the other paths are designed to support only the TE_0_ mode. The arms of the Y junctions are connected to the single-mode waveguide of the MZI and when an optical signal is launched at the stem of the first Y junction, the optical power is distributed among the arms and then appears at the MZI. For efficient mode conversion, the path difference between two unbalanced arms is kept ΔL and after the signals are recombined at the second Y junction effective mode conversion is achieved.

**Figure 3:**
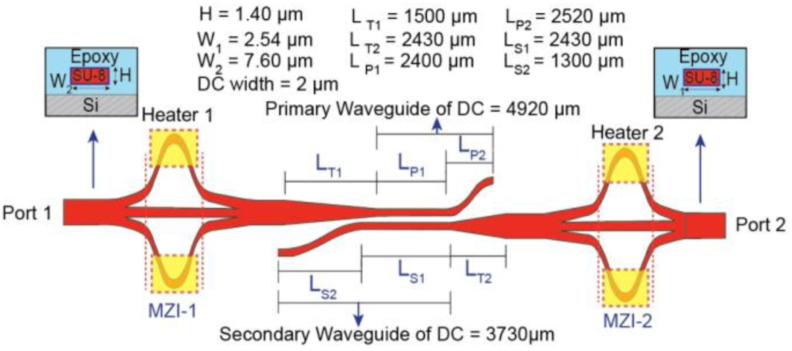
Cross-sectional view of the proposed structure.

In our design, we have used polymer materials for both the core and the cladding of the waveguides. To simulate the device performance as a high-order mode pass filter, we have used the Rsoft photonic computer-aided design suite 3D beam propagation method. We fix SU-8 2000 for the core material and epoxy OPTOCAST 3505 (Electronic Material Inc.) for the cladding and the refractive indices for the core and cladding materials are 1.57 and 1.512 respectively at 1.550 μm wavelength. In Figure 3 the waveguide thickness is set to H = 1.40 μm, the width of the single-mode region is fixed to W_1_ = 2.53 μm and the width of the three-mode region is W_2_ = 7.60 μm. We have used three-arm Y-junctions and to reduce the insertion loss, the half flare angle for each unbalanced arm is designed as 2.45°. The heating effect is simulated in our simulation tool by considering the thermo-optic effect of the core material and we assume for every °C increment the refractive index of the core reduces by 0.00008.

For coupling the power between the converters, we have preferred using the fundamental mode directional coupler (DC) to a straight single-mode waveguide to increase the extinction ratio (ER) of the device. The first taper (Taper 1) connects the mode converter 1 with the primary waveguide of the DC and the second taper (Taper 2) connects the secondary waveguide of the DC with mode converter 2 (Figure 2). The total length of the primary waveguide L_P_ (L_P1_+L_P2_) is 4920 μm while for the secondary waveguide L_S_ (L_S1_+L_S2_) is 3730 μm and the width of the waveguides of the DC is set to 2 μm. The distance kept between the waveguides of the DC is 1.50 μm to achieve a coupling efficiency of 99.73 % for the TE_0_-to-TE_0_ mode coupling. Finally, the total length of the device is 18.30 mm, which can be further minimized by using ultra-compact DC and taper designs.

**Figure 4:**
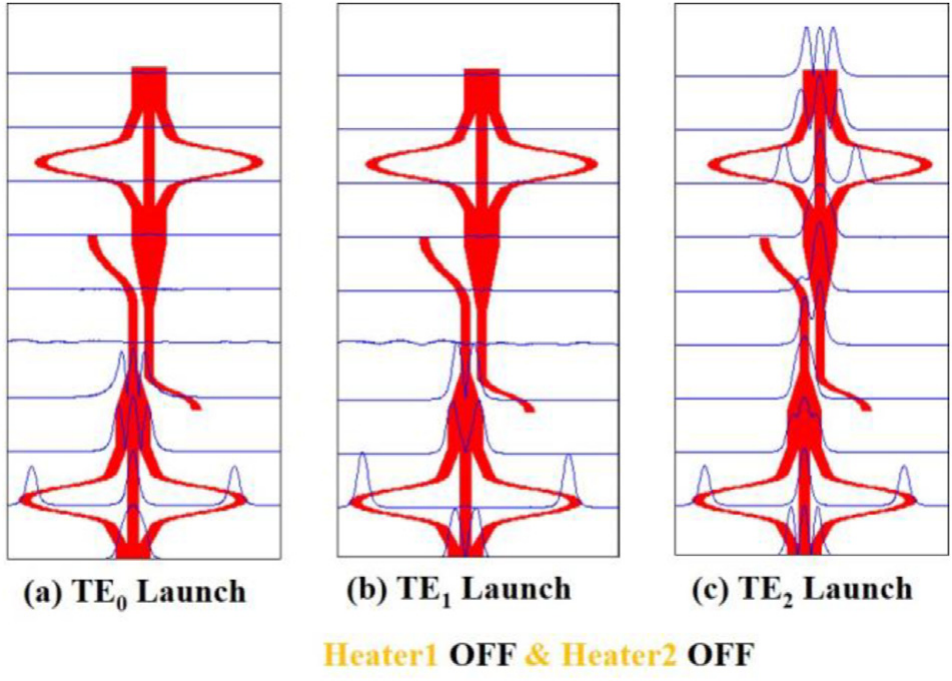
Simulation of mode filter device showing mode evolution at different positions for OFF-OFF condition of the two heaters using Beam propagation method when (a) TE_0_ mode (b) TE_1_ mode and (c) TE_2_ mode is launched at Port1.

**Figure 5:**
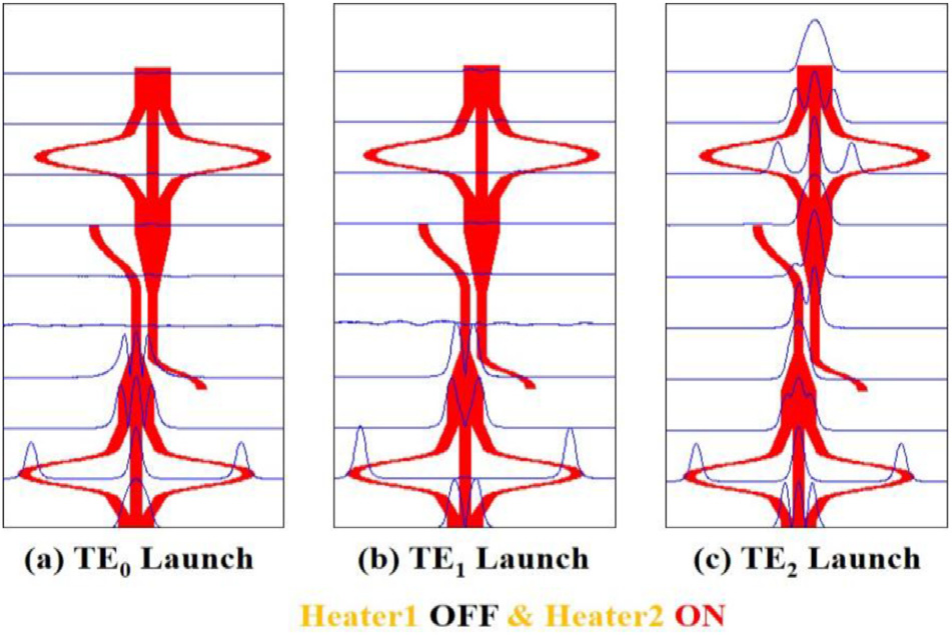
Simulation of mode filter device showing mode evolution at different positions for OFF-ON condition of the two heaters using Beam propagation method when (a) TE_0_ mode (b) TE_1_ mode and (c) TE_2_ mode is launched at Port1.

**Figure 6:**
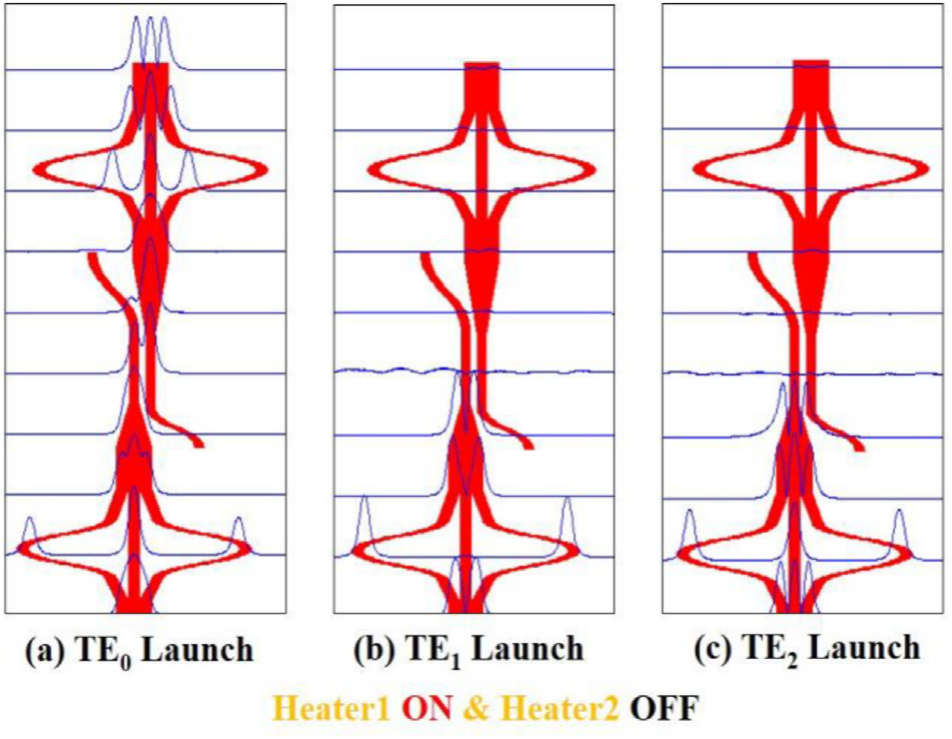
Simulation of mode filter device showing mode evolution at different positions for the ON-OFF condition of the two heaters using Beam propagation method when (a) TE_0_ mode (b) TE_1_ mode and (c) TE_2_ mode are launched at Port1.

**Figure 7:**
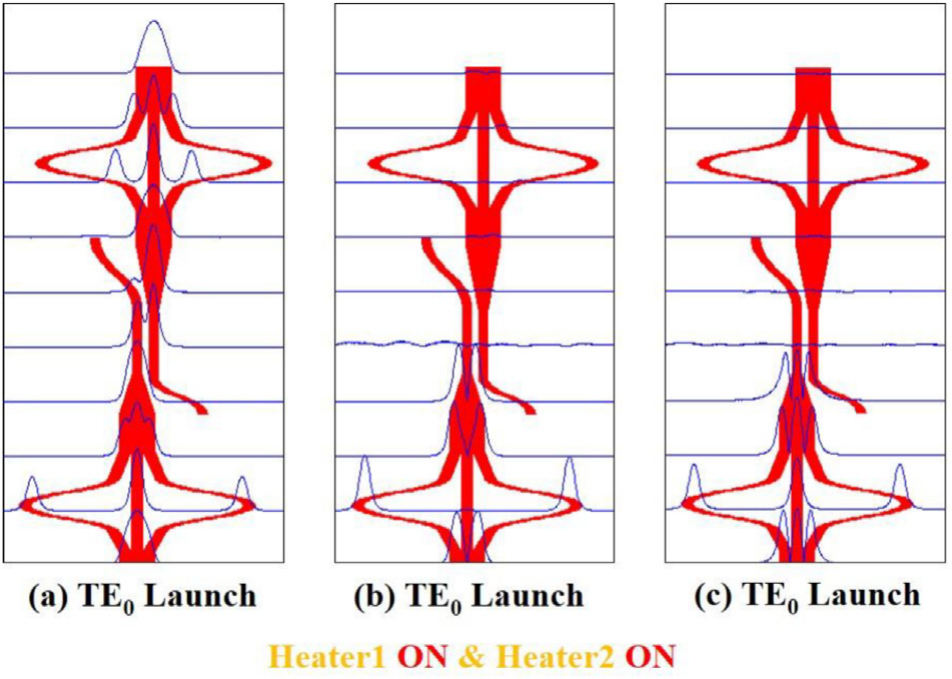
Simulation of mode filter device showing mode evolution at different positions for ON-ON condition of the two heaters using Beam propagation method when (a) TE_0_ mode (b) TE_1_ mode and (c) TE_2_ mode are launched at Port1.

From Figures 4, 5, 6, and 7, the operation of the mode filter can be easily realized by observing the propagation path of different modes at different sections of the device for different switching conditions. In Figures 4(a), 4(b), and 4(c) we can see that when both heaters are “OFF”. Hence, for the launched mode at the mode converter1, TE_0_ is converted to TE_2_ mode, and TE_2_ mode is converted to TE_0_ but the TE_1_ mode remains unchanged. Figure 4(a) shows, that the launched TE_0_ mode enters into the taper as TE_2_ and starts losing its power and then loses all its power at the primary waveguide of the DC.

In Figure 4(b) the TE_1_ mode is also radiated out in the same way. In both cases, no coupling occurs at the secondary waveguide of the DC and no power appears at the output port. Thus lower-order modes are filtered out. However, in Figure 4(c) the TE_2_ mode appears at the taper as TE_0_ mode and as the lower-order mode is well confined at the waveguide, no power is lost at the taper, moreover, the coupling starts at the secondary waveguide of the DC. More than 90% power is coupled at the secondary waveguide and then the coupled power appears at the mode converter 2 through a reverse taper. Finally, the TE_0_ mode is converted back to the TE_2_ mode at the output port which exhibits that the structure works as a higher-order mode pass filter. In Figures 5(a), 5(b), and 5(c) the first mode converter is “OFF” and the second mode converter is “ON”. The same phenomenon appears in Figure 5(a) and Figure 5(b) that as in Figure 4(a) and Figure 4(b), respectively. But the result in Figure 5(c) is different from Figure 4(c) and in this case, TE_0_ mode is output for launched TE_2_ mode. Again, in Figures 6(a), 6(b), and 6(c) the first mode converter remains “ON” and the second mode converter remains “OFF”. So, the launched TE_0_ mode passes the coupler spontaneously but the TE_2_ mode appears at the output of the second mode converter. On the other hand, Figures 7(a), 7(b), and 7(c) show that, when both heaters are in the “ON” state, the reverse operation happens when the mode converters are in the “OFF” state. It means in Figure 7(a), the TE_0_ mode appears at the coupler without any conversion, coupled at the DC, and passing through the second mode converter appears at the output port. Thus, the structure passes lower-order mode and the TE_1_ and the TE_2_ mode are filtered out respectively in Figures 7(b) and 7(c). Thus, the device function as the fundamental mode pass filter. Basically, which mode will be passed through the device depends on the first mode converter and the second mode converter is used to form the desired output mode.

## 3 Simulation and results

To demonstrate our simulation results of the mode filter, the term mode rejection and mode transmission are defined as:

(1) Mode rejection, (dB) = 10 log_10_ (P_out_)

For the undesired modes at the output port;

(2) Mode transmission, (dB) = 10 log_10_ (P_out_)

For the desired mode in the output port;

P_out_ = Normalized power at output port

**Figure 8:**
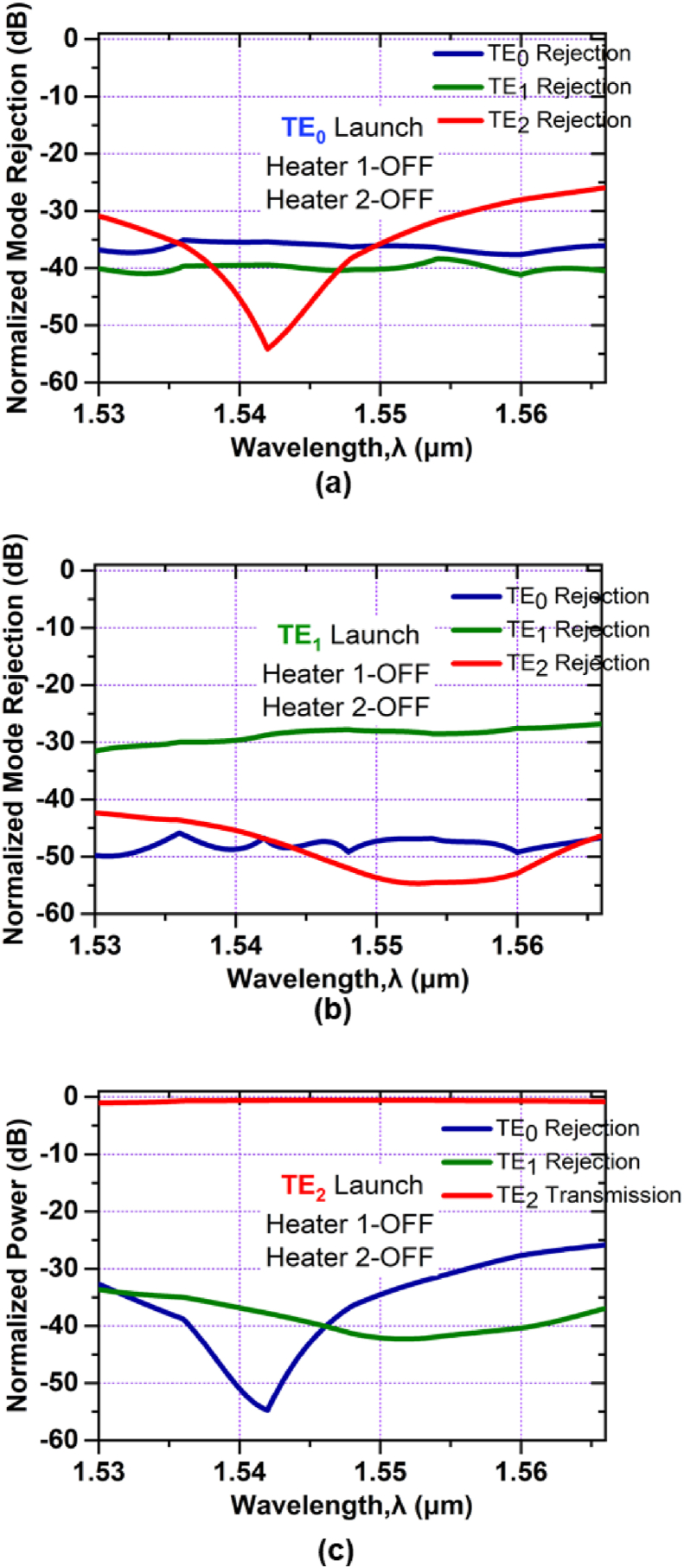
Simulated normalized mode power at Heater 1-OFF and Heater 2- OFF when (a) TE_0_ mode (b) TE_1_ mode and (c) TE_2_ mode is launched at Port1.

**Figure 9:**
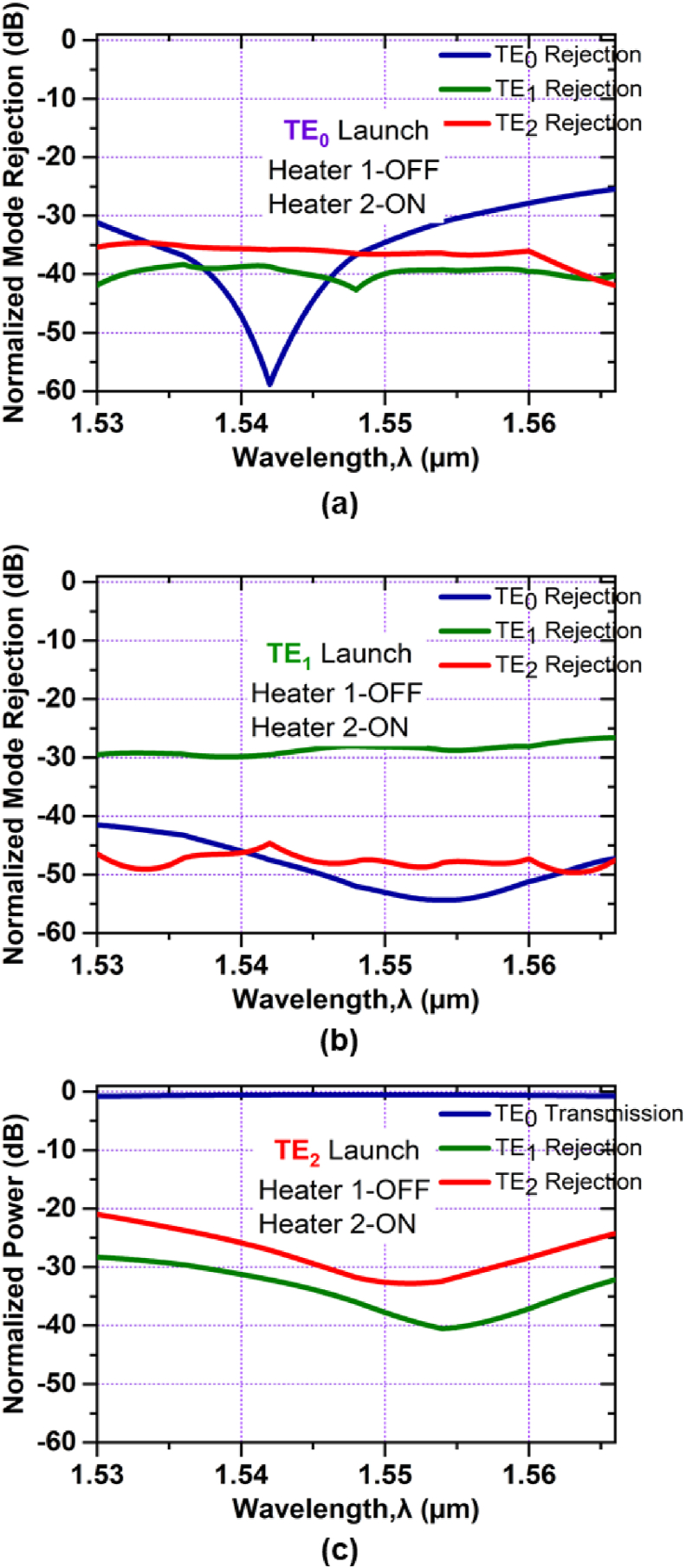
Simulated normalized mode power at Heater 1-OFF and Heater 2-ON when (a) TE_0_ mode (b) TE_1_ mode and (c) TE_2_ mode is launched at Port1.

For the case of a higher-order mode pass filter the normalized mode power at the output port for launching TE_0_, TE_1_, and TE_2_ mode respectively can be realized in Figure 8. In Figure 8(a) we can see that, the TE_0_ mode rejection is -36 dB and the TE_1_ mode rejection is -40 dB for the central wavelength of 1.550 μm and almost flat for the entire C band. At 1.550 μm, TE_2_ mode rejection is -36 dB and less than -26 dB for the whole C band. Again from Figure 8(b), the mode rejections for the TE_0_ and the TE_1_ are respectively -46 dB and -28 dB at wavelength 1.550 μm and for the TE_2_ mode, rejection is less than -42 dB. On the contrary, in Figure 8(c) we can see that, when the higher-order mode i.e. TE_2_ mode is launched, all the lower-order modes are rejected; with ∼35 dB rejection for TE_0_ mode and ∼43 dB rejection for TE_1_ mode at the central wavelength and only TE_2_ mode can pass through the filter with a small simulated excess loss of ∼0.61 dB for the entire C band. The device characteristics at Heater 1-OFF and Heater 2-ON conditions are shown in Figure 9.

As in Figure 9(a), for launching TE_0_ mode, the mode rejection for TE_0_ mode is -34 dB at 1.550 μm and less than -25 dB for the entire C band. For TE_1_, and TE_2_ modes, the mode rejections are -40 dB and -35 dB respectively at 1.550 μm and almost flat for the whole C band. For TE_1_ mode launching, Figure 9(b), the mode rejections for TE_0_, TE_1_, and TE_2_ modes are less than -42 dB, -27 dB and -45 dB respectively at the entire C band. When TE_2_ mode is launched, as shown in Figure 9(c), there is the conversion of TE_2_ mode to TE_0_ mode has a small simulated excess loss of ∼0.61 dB for the entire C band. The mode rejections for TE_1_ and TE_2_ modes are less than -37 dB and -33 dB respectively at 1.550 μm.

**Figure 10:**
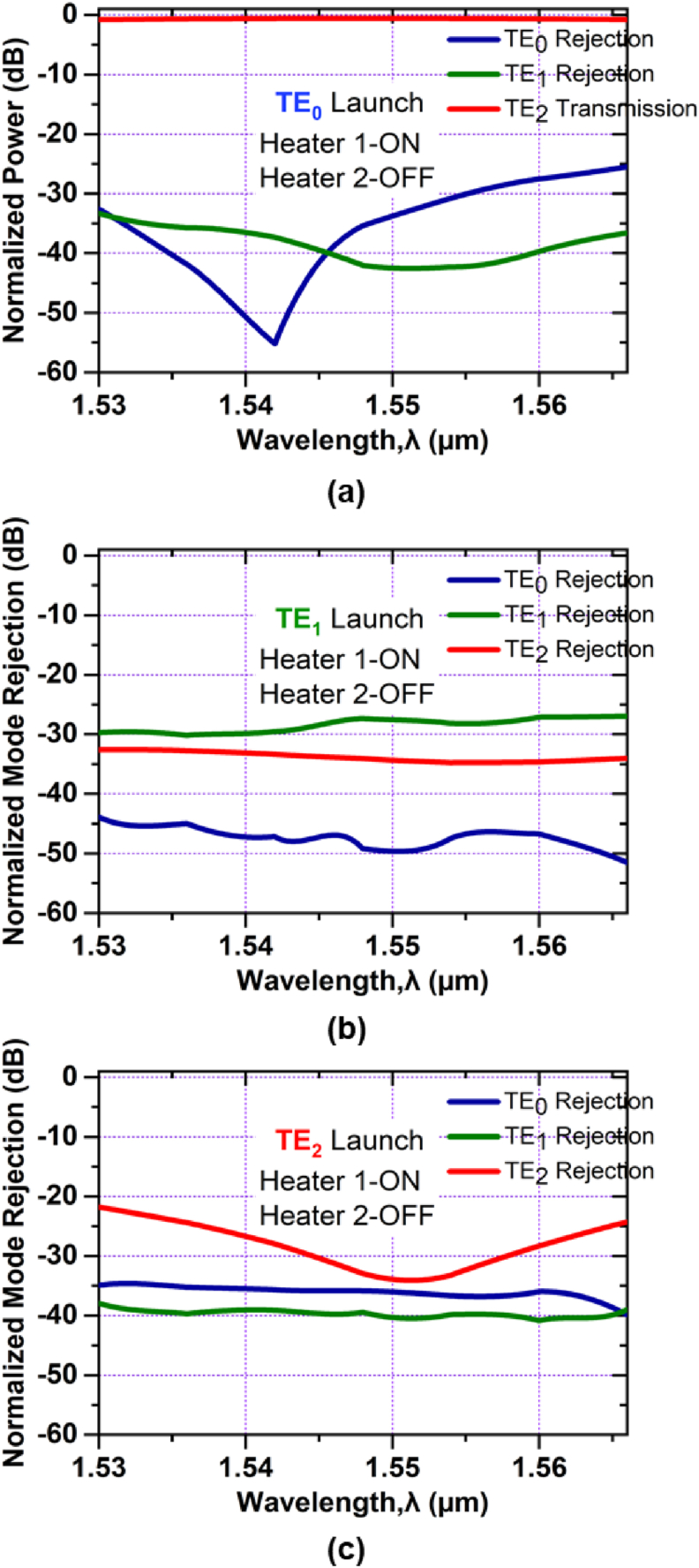
Simulated normalized mode power at Heater 1-ON and Heater 2-OFF when (a) TE_0_ mode (b) TE_1_ mode and (c) TE_2_ mode is launched at Port1.

For Heater 1-ON and Heater 2-OFF conditions, the device performance is given in Figure 10. As shown in Figure 10(a), the launched TE_0_ mode is converted to TE_2_ mode with simulated excess loss of ∼0.61 dB for the entire C band. TE_1_ and TE_2_ modes have mode rejections of less than -34 dB and -43 dB respectively at 1.550 μm. In Figure 10(b), the mode rejections for TE_0_, TE_1_, and TE_2_ modes are less than -44 dB, -27 dB and -33 dB respectively for the whole C band. For TE_2_ mode launching, Figure 10(c), the mode rejections are -36 dB, -40 dB and -34 dB for TE_0_, TE_1_, and TE_2_ modes respectively at 1.550 μm.

**Figure 11:**
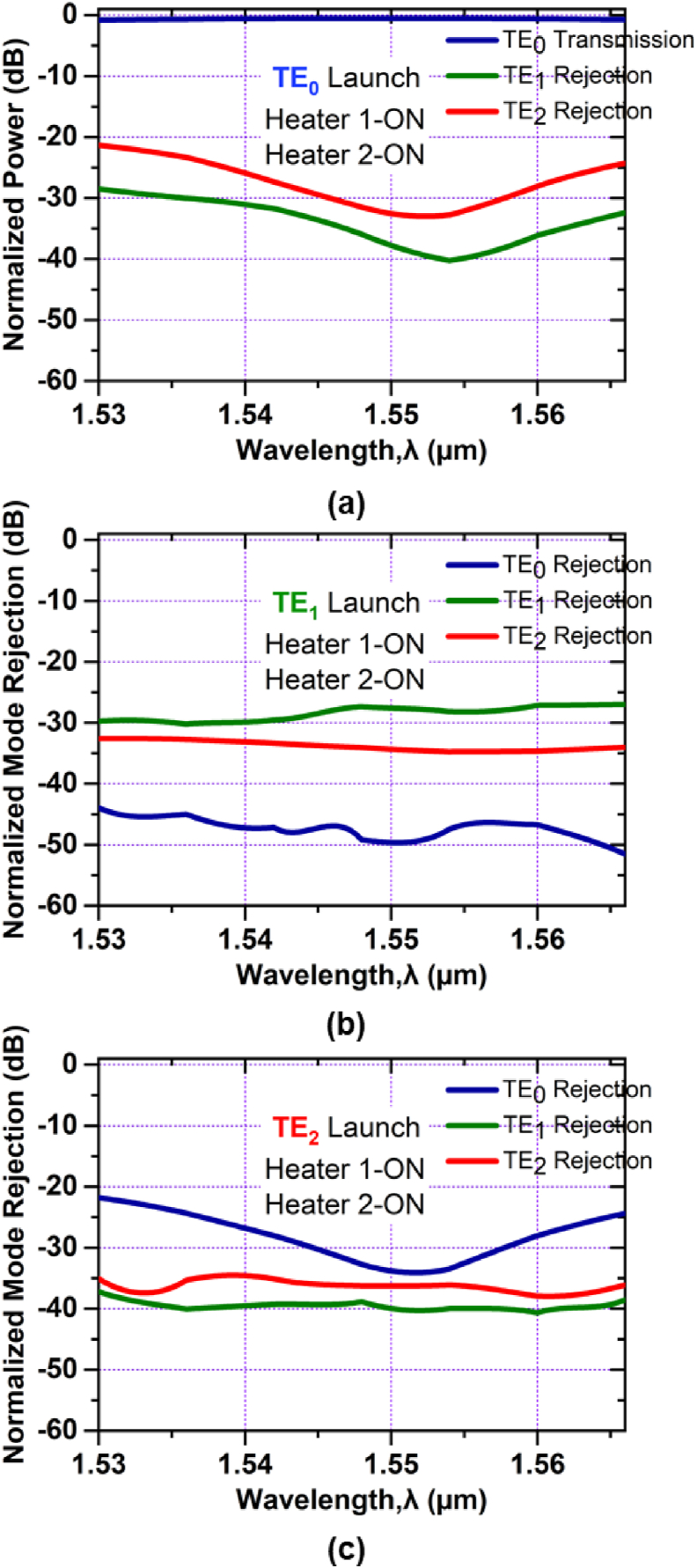
Simulated normalized mode power at Heater 1-ON and Heater 2- ON when (a) TE_0_ mode (b) TE_1_ mode and (c) TE_2_ mode is launched at Port1.

The device's performance as a fundamental mode pass filter is illustrated in Figure 11. When TE_0_ mode is launched, as shown in Figure 11(a), all higher-order modes are rejected; having -37 dB and -33 dB rejections for the modes of TE_1_ and TE_2_ respectively, at 1.550 μm and only mode TE_0_ can pass along the device possessing simulated excess loss ∼0.61 dB for the entire C band. The mode rejections, Figure 11(b), for TE_0_, TE_1_, and TE_2_ modes are lower than -44 dB, -26 dB and -33 dB respectively for the entire C band. For the TE_2_ mode launched, as in Figure 11(c), the mode rejections are -36 dB, -34 dB and -41 dB for TE_0_, TE_1_, and TE_2_ modes respectively at the center wavelength.

The majority of the reported mode filter devices are passive and cannot be actively tuned. Switchable mode filters have been reported rarely. T. Huang et al. [20] proposed a reconfigurable mode filtering technique based on phase transition in vanadium dioxide (VO2). However, in high order filtering mode, this device has a low extinction ratio (<16 dB). Furthermore, the device can only block one mode, whereas multiple mode filtering requires the cascading of various mode blocking filters. Ref [27] demonstrated an experimentally tunable two-mode filter based on mode conversion [26]. hence, our proposed reconfigurable mode filter device capable of processing higher optical modes is required for flexible MDM network to achieve full system capacity.

## 4 Tolerance analysis

Fabrication tolerance analysis is an important factor to realize the robustness of a design against manufacturing disturbances. This factor with a high tolerance limit is efficient in a modelling photolithography process to design an optimum device.

**Figure 12:**
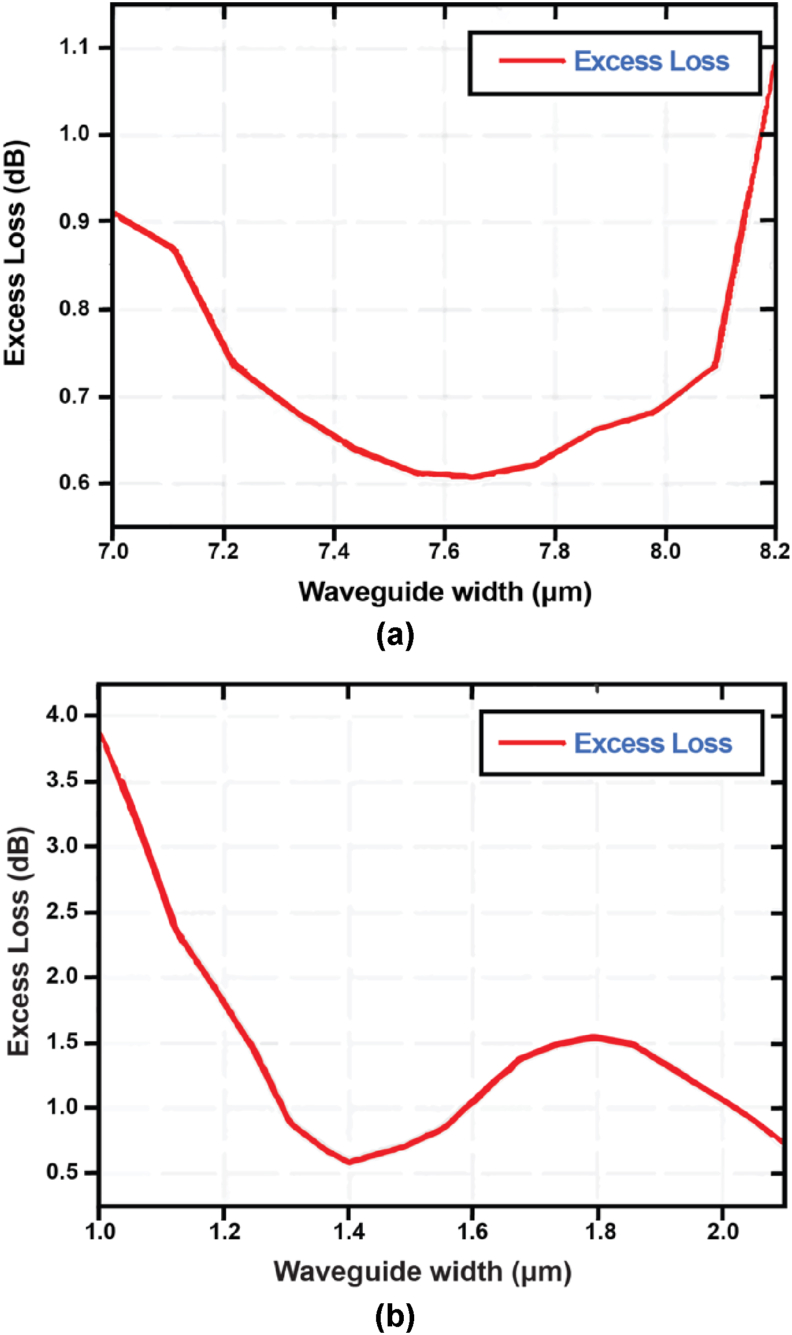
Variations of the excess loss with the variations of (a) Waveguide width and (b) Waveguide height.

In Figure 12 the variations of excess loss have been shown with varying waveguide width and height. In our proposed design we have used 7.60 μm as the waveguide width and 1.40 μm as the waveguide height. From Figure 12(a), we can see that at 7.60 μm the excess loss is minimum and from 7.50 μm to 7.70 μm there is a negligible change in the excess loss. At width ≤7.40 μm and ≥7.80 μm, the excess loss increases by ≥ 0.05 dB which means the device is less sensitive at this limit. Again in Figure 12(b), the excess loss is minimum at a height of 1.40 μm and if the height is reduced or increased by 0.15 μm the increase in excess loss is very small. But ±0.20 μm the excess loss increases rapidly which shows the high sensitivity of the design at higher heights. Moreover, this analysis shows the design has less sensitivity for width variations compared to height variations.

## 5 Conclusion

We have proposed a simple and flexible approach of mode filter for MDM technique which can be used as HOM pass filter as well as a fundamental mode pass filter optionally and the output modes are also controllable. The operation of this device is realizable for both the TE mode and the TM mode. The simulated result shows a high extinction ratio and low excess loss of ∼0.61 dB at the C-band which ensure good performance of our proposed structure. There's scope for increasing the flexibility of this structure by tuning TE_1_ mode. The operating principle of the device can be used to develop a higher-order mode filter of any order by simply replacing our designed mode converters. The same structure can be extended to realise mode filtering with an increased number of optical modes by integrating the directional coupler with the two switchable mode converters. The switchable property should be further explored by controlling the phase of the propagating modes in each arm of the extended mode filters thermo-optically. Further investigation on C + L band can add diversity in this work.

## Declarations

### Author contribution statement

Prapty Saha; Kazi Tanvir Ahmmed: Conceived and designed the study; performed the simulation and the acquisition of data; wrote the paper.

Oruni Aminul; Md. Atiqur Rahman; Md. Shah Alam: Analyzed and interpreted the data; wrote the paper.

### Funding statement

This work was supported by the Research and Publication Cell, University of Chittagong [201/2020].

### Data availability statement

Data included in article/supp. material/referenced in article.

### Declaration of interests statement

The authors declare no conflict of interest.

### Additional information

No additional information is available for this paper.
